# Circadian dependency of microglial heme oxygenase-1 expression and inflammation determine neuronal injury in hemorrhagic stroke

**DOI:** 10.1186/s12950-023-00371-w

**Published:** 2023-12-16

**Authors:** Luise Henrich, Iva Kiessling, Matti Steimer, Sibylle Frase, Sandra Kaiser, Nils Schallner

**Affiliations:** 1 Department of Anesthesiology & Critical Care, Medical Center, University of Freiburg, Hugstetter Str. 55, Freiburg, 79106 Germany; 2 Faculty of Medicine, University of Freiburg, Freiburg, Germany; 3 Department of Neurology and Neuroscience, Medical Center, University of Freiburg, Freiburg, Germany

**Keywords:** Circadian rhythm, Carbon Monoxide, Heme Oxygenase, Microglia, Phagocytosis, Brain Hemorrhage, Neuroinflammation

## Abstract

**Background:**

The heme oxygenase-1 (HO-1) enzyme pathway is of crucial importance in the removal of toxic blood components and regulation of neuroinflammation following hemorrhagic stroke. Although a circadian pattern dependency in the incidence and severity of hemorrhagic stroke exists, it is unknown whether the activity of the HO-1 system in the context of hemorrhagic injury also exhibits circadian dependency. We hypothesized that the circadian regulation of microglial HO-1 would determine the extent of neuroinflammation and neuronal injury in a murine model of subarachnoid hemorrhage (SAH).

**Methods:**

In vitro expression patterns of *HO-1* and circadian rhythm genes were analyzed in the microglial BV-2 cell line and primary microglia (PMG) using Western blot and qPCR. PMG isolated from *Hmox1*^*fl/fl*^ and *LyzM-Cre-Hmox1*^*fl/fl*^ mice were used to evaluate the role of microglial HO-1. We further investigated the in vivo relevance in a murine subarachnoid hemorrhage (SAH) model using *Hmox1*^*fl/fl*^ and *LyzM-Cre-Hmox1*^*fl/fl*^ mice with myeloid cell *HO-1* deficiency, inducing SAH at different zeitgeber (ZT) times and analyzing the expression of *HO-1* and the circadian control gene *Period-2 (Per-2*), respectively. Furthermore, we measured the inflammatory cytokine Monocyte Chemoattractant Protein-1 (MCP-1) in the cerebrospinal fluid of SAH patients in correlation with clinical outcome.

**Results:**

*HO-1* baseline expression and response to CO with blood exposure depended on ZT. In vitro expression of circadian control genes was de-synchronized in *LyzM-Cre-Hmox1*^*fl/fl*^ PMG and did not respond to exogenous CO exposure. We found that circadian rhythm plays a crucial role in brain damage after SAH. At ZT2, we observed less phagocytic function, more vasospasm and increased microglial activation. CO reduced mortality at ZT12 in HO-1 deficient mice and reduced the difference between ZT2 and ZT12 in the inflammatory response. Induction of MCP-1 in the CSF from SAH patients was time-dependent and correlated with the expression of circadian control genes, SAH severity, functional impairment and delirium.

**Conclusions:**

Our data point towards a crucial role for the HO-1 enzyme system and circadian control in neuronal injury after a hemorrhagic stroke.

**Supplementary Information:**

The online version contains supplementary material available at 10.1186/s12950-023-00371-w.

## Background

The incidence and severity of subarachnoid hemorrhage (SAH), a subtype of hemorrhagic stroke, follow a typical circadian pattern with a peak of cases during the morning hours [1]. Although this is a well-described epidemiological phenomenon, a pathophysiological explanation apart from diurnal physical activity is still missing [2].

SAH occurs in 10 per 100,000 patients per year [3, 4] and leads to neuronal injury via heme-mediated cerebral inflammation and blood component toxicity. Elimination of heme usually occurs through the heme oxygenase (HO) enzyme pathway that degrades heme to biliverdin, iron and the gas carbon monoxide (CO). Of the two isoforms, induction of HO-1 protects several organs including visceral organs [5–7], the heart [8], and the central nervous system [9–14] from apoptotic processes post injury. HO-1 possesses potent anti-inflammatory properties [15], which is of importance for the regulation of the neuroinflammatory response after SAH. We have recently identified novel roles for HO-1 in microglia in response to SAH [16]. Microglial *HO-1* expression itself mediates the clearance of blood and its produced gaseous molecule CO in turn regulates erythrophagocytosis [17, 18].

Circadian rhythm is essential for every living organism and determines the physiological function of nearly every organ [19]. Disruption of the normal circadian cycle is common in critically ill patients such as patients with intracranial bleeding and it influences clinical outcomes [20]. Circadian rhythm in the whole organism is coordinated in the suprachiasmatic nucleus (SCN) which is located in the hypothalamus. On the molecular level, circadian rhythmicity relies on an evolutionary well-conserved transcriptional feedback mechanism involving transcription factors (*CLOCK*, *BMAL-1, RevErb-α* and *NPAS-2*) that control the expression of up to 43% of the whole genome [21, 22] and regulatory proteins including *Period (Per) 1–3* and *Cryptochrome (Cry) 1–2*. Experimental data suggest a direct role of these regulatory proteins and transcription factors in cardiovascular [23] and neuronal disease [24] besides circadian regulation. Interestingly, a direct link has been established between the HO-1-CO enzyme system and circadian regulation: CO as the product of HO-1-mediated heme metabolism can directly influence the transcriptional activity of *NPAS-2* [25] and *CLOCK* [26].

Indeed, we have previously demonstrated in mice that CO can restore circadian rhythmicity in the CNS and peripheral organs after hemorrhage [18], which critically influences the severity of neuronal injury. Also, we discovered that patients show altered expression of HO-1 [27] and genes controlling circadian rhythm [28] in cerebrospinal fluid following SAH. In light of these findings, the influence of the HO-1-CO system regarding circadian regulation and its influence on the extent of neuronal injury after SAH remains to be further explored. Therefore, we hypothesized that circadian rhythm influences HO-1 expression, reactivity to therapeutic CO application, HO-1-dependent microglial function and the associated neuroinflammatory response in a murine SAH model as well as SAH patients.

## Materials and methods

### Animals and anesthesia

Cell-type-specific HO-1 knockout in myeloid cells including microglia (*LyzMCre-Hmox1*^*fl/fl*^) was achieved by crossing *Hmox1*^*fl/fl*^ mice (Riken Bio Resource Center, RBRC03163) with mice expressing Cre recombinase under the lysozyme (Lyz) promoter (Jackson Laboratory, #004781). *Hmox1*^*fl/fl*^ microglia were used as controls for all in vitro studies. Animals were fed with a standard rodent diet *ad libitum* while kept on a 12-h light/12-h dark cycle. All types of surgery and manipulations were performed under general anesthesia with ketamine (100 mg/kg) and xylazine (5 mg/kg) and body temperature maintenance. After surgery, buprenorphine (50 µg/kg) was applied subcutaneously to treat possible pain.

### Subarachnoid hemorrhage (SAH) model

SAH was achieved by pre-chiasmatic injection of blood withdrawn from the hearts of donor mice. Only male mice were used for the SAH treated mouse group as well as the donor mice.After induction of anesthesia, the head was fixed in a stereotactic frame. The anterior skull was exposed with a midline skin incision. 3.5 mm anterior to the bregma, a 0.8 mm burr hole was drilled into the skull with a caudal angle of 40°. A 27-G needle was advanced through the burr hole at a 40° angle until the base of the skull was reached, then 40 µl of whole blood was slowly injected and the needle left in place for 1 min to avoid backflow. After observing for overt bleeding, the skin was sutured, and animals were allowed to emerge from anesthesia under close supervision with body temperature maintenance using infrared light. The mice were treated with buprenorphine (50 µg/kg) three times a day for the first three days postoperatively. SAH was either induced at ZT2 or ZT12, which corresponds to the end of the 2nd and the 12th hour of the 12-hour light cycle. We chose ZT2 and ZT12 as the time points of SAH because these correspond to the nadir and peak within the circadian gene expression profile.

### CO gas treatment

After SAH, animals were randomly assigned to either receive treatment with CO or air for 1 h. CO exposure was done in a custom-made chamber. Animals had free access to food and water during the treatment. Pre-mixed air with 250 ppm CO was used. Treatment was started immediately after the SAH and repeated every 24 h for 1 to 7 days, depending on the specified readouts.

### Barnes maze behavioral studies

Spatial learning was tested on the Barnes maze. In brief, the paradigm consisted of a white circular platform with a diameter of 120 cm and 40 equal-sized holes along the perimeter. Beneath one hole, a box into which the animals could escape from the open platform was positioned. As an aversive stimulus to leave the platform, a bright light source was placed above the setup. A rewarding stimulus was implemented by placing food pellets into the escape box. Cardboard symbols with different visual cues were placed around the platform as orientation marks. During the initial acquisition period, the location of the escape box and the visual cues were kept constant. The mice underwent initial spatial acquisition for 7 days with 3 trials per animal per day. A maximum of 3 min for exploration of the maze was allowed per animal. Thorough cleaning of the platform after each animal to eliminate olfactory cues was performed. Animals who failed to enter the escape box within 3 min were guided to the correct hole. The total time required for the mouse to find the goal box (latency) and the number of holes the animal explored before finding the goal box (error number) were measured as surrogates for cognitive function. SAH was induced after 7 days of acquisition. Spatial memory testing consisted of 1 trial per animal per day starting on day 1 after SAH until day 7 after SAH. To test for flexibility and relearning, the location of the goal box was changed to the exact opposite position on day 4 (spatial reversal) with all visual cues kept constant. Therefore, relative latency times in the Barnes maze spatial memory paradigm for functional outcome in the results from day 14 were measured 6 days after SAH induction and 2 days after spatial reversal.

### Hematoxylin/eosin staining and evaluation of cerebral vasospasm

7 days after SAH, animals were deeply anesthetized with ketamine and xylazine and transcardially perfused with TBS followed by PFA 4%. Brains were removed and fixed in PFA 4% for 18 h. After cryoprotection in sucrose, brains were frozen, cut into 8 μm sections and stained with hematoxylin and eosin. Representative digital images of 2–3 consecutive middle-cerebral artery (MCA) cross-sections from each animal were obtained and the lumen radius/wall thickness ratio was quantified to assess vasospasm using Image J. The outer and inner vascular circumferences were measured with Image J. The vasospasm index was calculated by inner circumference divided by de difference of outer circumference minus inner circumference.

### Immunohistochemistry

Frozen brains were cut into 8 μm serial coronal sections. The glass slides with brain sections were heated in 1x citrate buffer pH 6 (Zytomed System GmbH, K035) in a microwave (3 × 5 min 800 W). Permeabilization was done with 0.1% Triton/PBS for 10 min at room temperature (Iba-1) or with 0.2% Tween/PBS for 10 min at room temperature (TER-119 co-staining with Iba-1). Slides were then blocked in 10% donkey serum/PBS (Iba-1 or 4% BSA/PBS (TER-119 co-staining with Iba-1) for 1 h at room temperature. Staining was performed with primary antibodies against Iba-1 (Abcam AB5076, 1:200) or against TER-119 (Abcam AB91113, 1:100) with Iba-1 at 4 °C overnight. Sections were then conjugated with the corresponding secondary antibody for fluorescent imaging (for Iba-1 anti-goat Alexa Fluor 488, Abcam AB150129 and for TER-119 anti-rat Alexa Fluor 555, Abcam AB150154; 1:300). Nuclear counterstain was done with DAPI and slides were examined under a fluorescence microscope (Zeiss AxioObserver Invert). To achieve appropriate consistency in the quantification of cells, from each area of interest, 2–4 images were obtained of 3–4 mice per group. For analysis of microglia phagocyting erythrocytes, cells with colocalization of TER-119 and Iba-1 were counted. Activated microglia are characterized by larger cell bodies with retracted extensions while resting microglia show smaller cell bodies with extended ramifications. Therefore, we measured the soma size of Iba-1 positive microglia in coronal brain sections and used the mean of soma sizes as a hallmark of activation by area with ZEN 2.5 (blue edition) software from Carl Zeiss Microscopy. For representation in the figures, larger analyzed images were cropped down to one representative area, cell or vessel per group.

### BV-2 and PMG cell culture and treatment

The feasibility of circadian studies in vitro has been shown before with robust in vitro expression of genes relevant to circadian control and the possibility of inducing these genes via various stimuli [29]. Circadian rhythm can be restored with adequate entrainment stimuli such as medium change [29]. Therefore, experiments were performed 2 and 12 h after the medium change. Matching resulting gene expression profiles with profiles from prior research resulted in the identification of an early and late phase of the circadian cycle [30, 31]. Early and late phases in vitro are corresponding to ZT2 and ZT12 in vivo.

BV-2 microglia cells were incubated in DMEM containing 1% penicillin-streptomycin and 10% FBS in a humidified atmosphere with 5% CO^2^. Cells were seeded into 6-well plates (western blot analysis) or 24-well plates (other experiments) at a density of 200,000 for individual experiments. PMG were isolated from *LyzM-Cre-Hmox1*^*fl/fl*^ or *Hmox1*^*fl/fl*^ mice at P5 to P7 (50% female and 50% male) by enzymatic neural dissociation (Papain Neural Dissociation Kit; Miltenyi Biotec) and in vitro mixed glia culture. Mouse brains were enzymatically dissociated according to the manufacturer’s instructions and the resulting mixed glia culture containing astrocytes and microglia was cultivated in DMEM containing 1% penicillin-streptomycin, 10% FBS, and M-CSF (10 ng/ml) in a humidified atmosphere with 5% CO2. After one full medium change and 1 week of cultivation, microglia were collected by shaking the cell culture plates at 200 rpm for 30 min every 2 to 3 days. Floating microglia were collected from the supernatant and seeded onto 24-well plates for experiments at a density of 2 × 105 cells per well. The microglial phenotype was confirmed by CD11b staining and flow cytometry (99,5% positive).

### Flow cytometry

Cells were harvested with a cell lifter, washed with flow cytometry buffer (PBS w/1% BSA, 2mM EDTA, 0.05% Na-Azide) and incubated with CD16/32 antibodies to block unspecific Fc-binding loci. Specific staining was performed with a PE-Cy7-tagged CD11b antibody (Biolegend, 1:100, 30 min). Flow cytometry was done on a FACS LSR Frida (B&D Biosciences) with a selection of CD11b positive PMG and detection of phagocytosis-related fluorescence in FL-1. The relative % of RBC-positive PMG and the mean fluorescence per PMG was calculated using FlowJo.

### Western blot analysis

Cells were washed with PBS and lysed in radioimmunoprecipitation assay (RIPA) buffer with PhosStop (Sigma Aldrich 4,906,845,001) and protease inhibitor (Sigma Aldrich 4,693,116,001) shaking at 1000 rpm for 10 min at 4 °C. Equal amounts of protein (50 µg) were separated on a 10% SDS (Sigma-Aldrich R0278) polyacrylamide gel (TGX Stain-Free™ FastCast™ 161–0183 Bio-Rad) and transferred to a polyvinylidene difluoride (PVDF) membrane (Trans Blot Turbo Transfer Pack 1,704,157 Bio-Rad) with a Trans Blot Turbo Transfer System (Bio-Rad). Pictures of the membrane were recorded under UV light for total protein display. Total protein was used as loading control to ensure higher comparability and better quantification. Membranes were blocked with 5% skim milk in 1X-TBS-T (Tris Buffered Saline with 20% Tween) and incubated in the recommended dilution of an antibody against HO-1 (Abcam: HO-1 AB52947, 1:1,000) overnight at 4 °C. Membranes were then incubated with the corresponding secondary antibody 1:5000 for chemiluminescence detection and developed on a Fusion FX imaging system with Western Lightning Plus-ECL (Perkin Elmer NEL103001EA). The photometrical analysis provided specific protein levels as well as total protein levels. Additional File 1 provides the raw image data for Western blot analyses.

### Gene expression analyses from cells and tissue samples

Cells were harvested by in-well lysis using a Trizol reagent. Tissue samples were immediately homogenized in Trizol and further processed for RNA purification. RNA concentration and purification were done using spin columns (RNEasy mini kit, Qiagen) according to the manufacturer’s protocol. cDNA templates were acquired by reverse transcription (iScript cDNA synthesis kit, BioRad). Gene expression of circadian rhythm genes was then analyzed by real-time PCR (SYBR green master mix, Agilent Technologies) with *Rplp0* as a reference gene.

Primer sequences are:

1. *Per-2*.

Forward: AGGATGTGGCAGGTAACAGG.

Reverse: CGTAAGGGAACACACTGAGAGG.

2. *Cry-1*.

Forward: TGAGAAATATGGCGTTCCTTCC.

Reverse: GTAAGTGCCTCAGTTTCTCCTC.

3. *Rplp0*.

Forward: GAGGAATCAGATGAGGATATGGGA.

Reverse: AAGCAGGCTGACTTGGTTGC.

### CBA assay

PMG were incubated with blood +/-CO at the indicated concentration and time. Cell supernatant was harvested to be analyzed using bead-based flow cytometry as the manufacturer instructed (BD 552,364, Mouse Inflammation Kit). The following targets were measured on a BD Fortessa flow cytometer: IL-6, IL-10, MCP-1, IFN-γ, TNF and IL-12p70. Data were analyzed with FCAP Array™ Software.

### SAH patient data and clinical study design

A total of 66 patients with aneurysmal SAH (19 male, 47 female, age 58.03 ± 13.22 years, range 27–88 years) admitted to either the Intensive Care Units of the Department of Neurology and Neuroscience or the Department of Neurosurgery at the University of Freiburg (Germany) Medical Center between 2017 and 2020 were included in this study. This patient collective represents a subpopulation analysis of a concurrent study with circadian gene expression and chemokine data being a secondary endpoint [27]. An a priori power analysis for the primary endpoint (Wilcoxon-Mann-Whitney-Test, CSF *HO-1* expression vs. mRS at discharge favorable/non-favorable, effect size Cohen’s w 0.8; α = 0.05; power 95%, df 2) revealed a necessary sample size of 54 patients for biomarker characterization.

The following criteria for inclusion were applied:

- Age > 18 years.
- aneurysmal SAH confirmed on CT scan or via lumbar puncture/CSF xanthochromia.
- Admission to the ICU and placement of an external ventricular drain (EVD) for therapeutic purposes as well as first CSF and blood sample collection within 24 h after the SAH event.
- Provision of informed consent from the patient, legal guardian or by proxy.

The following criteria for exclusion were applied:

- Age < 18 years.
- Admission later than 24 h after symptom onset.
- Current pregnancy.
- Death within 24 h of ICU admission.
- Evidence of septic aneurysm origin or evidence of ventriculitis/meningitis during the time period of sample collection.
- Evidence of subdural/epidural hematoma on initial CT imaging.

CSF and blood samples were obtained from SAH patients on day 1, day 7 and day 14 after SAH symptom onset. Blood samples were obtained from either arterial or central venous catheters, while CSF samples were acquired under sterile conditions from external ventricular drains placed for therapeutic purposes. Blood samples were stored at -80°C in RNA stabilizing reagent tubes (Tempus Blood RNA Tube, AB#4342792). RNA isolation from leukocytes was performed using the correspondent spin-column RNA isolation kit (Tempus Spin RNA Isolation Kit, AB#1710145). RNA content and purity were assessed photometrically (NanoDrop 2000 Spectrophotometer, Thermo Fisher Scientific Inc.). RNA from CSF cells was isolated with Trizol and concentrated by spin-column purification (RNeasy Micro Kit, Qiagen, Hilden, Germany). The reverse transcriptase PCR technique (iScript cDNA Synthesis Kit, BioRad#1708890; PeqStar 96 Universal Gradient, PeqLab) was used to reversely transcribe RNA into cDNA. Semi-quantification of cDNA was performed via real-time PCR (sqPCR; StepOnePlus Real-Time PCR-System, A&V Applied Biosystems) with nucleic acid stain (PowerUp SYBR Green Master Mix, AB#1708020) and specific primers for *HO-1* and *Rpl13a* (ribosomal protein L13a, serving as intra-individual reference gene). CSF and blood samples of patients without intracranial hemorrhage (patients receiving shunt surgery for normal pressure hydrocephalus) served as inter-individual reference population to calculate relative changes in mRNA expression levels. Expression levels of *HO-1* mRNA were quantified using the 2^−∆∆Ct^” (cycle threshold) method, where ∆Ct = Ct (target gene *HO-1*) – Ct (reference gene *Rpl13a*) and ∆∆CT = ∆CT (study population) - ∆CT (reference population). Primer Sequences were:

**Table:** 

*HO-1* forward	GTGATAGAAGAGGCCAAGACTG
*HO-1* reverse	GAATCTTGCACTTTGTTGCTGG
*PER-2* forward	TCCTCGGCTTGAAACGGC
*PER-2* reverse	GAACGAAGCTTTCGGACCTCA
*Rpl13a* forward	CGGACCGTGCGAGGTAT
*Rpl13a* reverse	CACCATCCGCTTTTTCTTGTC

The following clinical parameters were collected: SAH severity scores (Hunt & Hess grade, modified Fisher grade, WFNS (World Federation of Neurosurgical Societies) grade), the occurrence of delirium (NuDESC score), modified Rankin Scale (mRS) at admission, discharge and one year after discharge, SAH hematoma volume (semi-quantitative Hijdra sum score on the initial CT scan [32]. Investigators performing clinical and radiographic ratings were blinded regarding laboratory results (gene expression, CBA assay) and vice versa.

For chemokine analysis from patients’ CSF and serum, a bead-based flow cytometry kit was used as the manufacturer instructed (BD 552,990, Human Chemokine Kit). The following targets were measured on a BD Fortessa flow cytometer: IL-8, RANTES, MIG, MCP-1 and IP-10. Data were analyzed with FCAP Array™ Software.

### Statistics

Data were analyzed with a computerized statistical program (GraphPad Prism Version 8). Results are presented as means (± SD). Two groups were compared with Student’s t-test and multiple groups were compared with either one-way or two-way ANOVA with post hoc Tukey multiple comparisons. For relative comparison vs. an averaged control group, one-column statistics with the t-test was used. To reduce confounding factors, we calculated relative changes within the data collected on the same day. Mathematically this resulted in the reference population being “1” for in vitro data. Therefore, one-sample t-tests against 1 were used to analyze those data. For better visualization, the reference group is indicated by a line at 1 when the shown data was normalized. Spearman non-parametrical correlation (rs, p), as well as linear regression (r^2^, *P*), was used to assess the correlation between metrical data sets (hematoma volume, *HO-1/Per-2* expression, MCP-1 concentration, modified Rankin Scale, WFNS grades). Survival curves were analyzed by the Mantel-Cox test. A *p*-value smaller than 0.05 was considered statistically significant.

## Results

### HO-1 protein expression levels depend on circadian timing

**Fig. 1:**
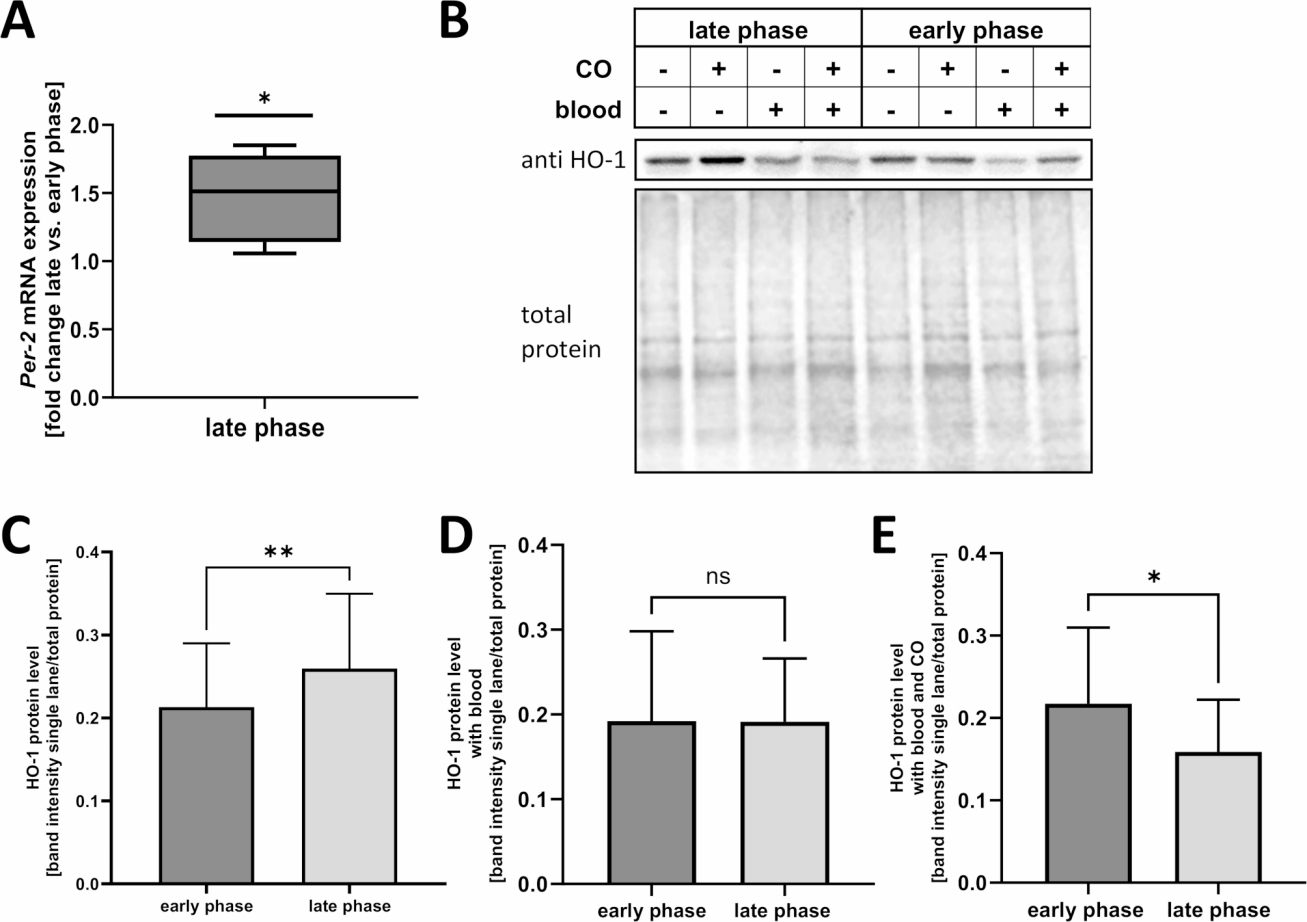
**(A-E):** Circadian difference in HO-1 protein expression in BV2 cells **(A)**  *Per-2* mRNA level in microglia cell line BV2 in the late and early phase of the circadian cycle demonstrated as fold change late vs. early phase. Early and late phases in the circadian cycle were induced by different timing of the medium change. (early vs. late phase *p* = 0.0247, n = 5). **(B-D)** Western blot showing time-dependent HO-1 protein expression level with or without carbon monoxide (CO) and blood exposure in the microglia cell line BV2. Shown late phase on the left, early phase on the right. Early and late phases in the circadian cycle were induced by different timing of medium change, treatment with 250 ppm CO and/or 100 µl blood for 1 h (h). **(B)** Representative western blot. **(C)** Western blot quantification of HO-1 protein expression level early vs. late phase in BV2 cells, *p* = 0.0197, n = 5. **(D)** Western blot quantification of HO-1 protein expression level in BV2 cells treated with 100 µl blood for 1 h.early vs. late phase, *p* = 0.9822, n = 5. **(E)** Western blot quantification of HO-1 protein expression level in BV2 cells treated with 100 µl blood and 250 ppm CO for 1 h early vs. late phase, *p* = 0.0128, n = 5, Used statistical analysis: paired t-test; results were presented as box blot with median, minimum and maximum or bars of means with SD. Statistically significant values were defined as *p* ≤ 0.05 (* *p* ≤ 0.05; ** *p* ≤ 0.01, *** *p* ≤ 0.001, **** *p* ≤ 0.0001)

To explore potential circadian rhythmicity in HO-1-expression, we performed Western Blot analysis in the microglial cell line BV2. Medium change at different time points was used as a circadian rhythm restoration stimulus. Early and late circadian rhythm phases could be identified by different mRNA levels of *Per-2* (Fig. 1A).The cells were exposed to blood with and without CO treatment. Here we demonstrated a time-dependent increase in HO-1 protein expression in the late phase of the circadian cycle (Fig. 1B, C and *p* = 0.0197). Microglia exposed to blood loose the circadian dependency of HO-1 expression (Fig. 1D *p* = 0.9822). Treatment with exogenous carbon monoxide during blood exposure lead to a lower HO-1 expression in the late phase compared to the early phase (Fig. 1E *p* = 0.0128), thus indicating a higher inducibility in response to CO when comparing the data to the baseline data in Figure C.

### Lack of HO-1 leads to circadian desynchronization in microglia

**Fig. 2:**
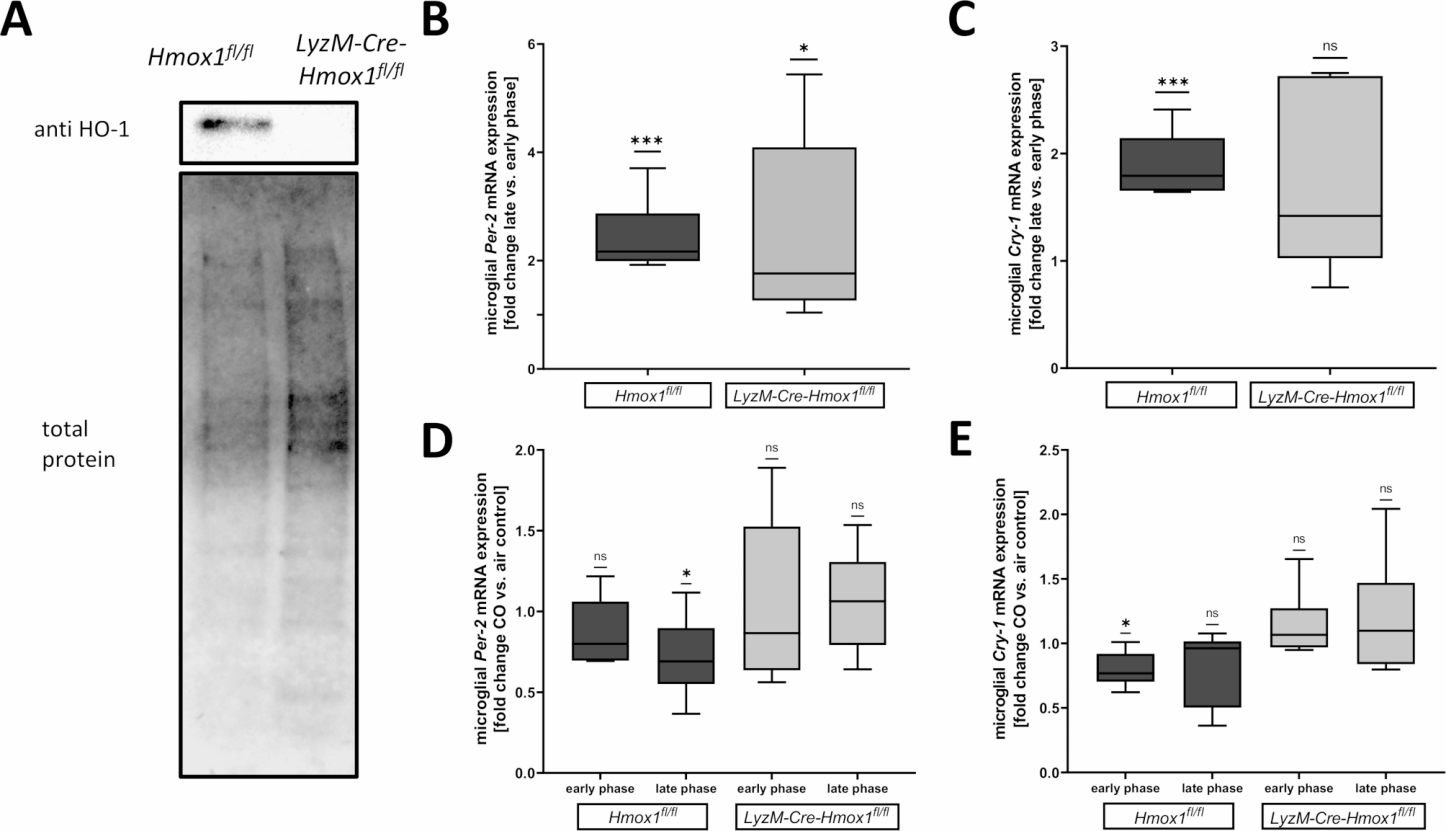
**(A-E):** Circadian difference in gene expression profiles in primary microglia **(A)** Western blot as proof of HO-1 knockout efficiency in *LyzM-Cre-Hmox1*^*fl/fl*^ compared to *Hmox1*^*fl/fl*^ cultured primary microglia cells. The lower panel shows corresponding loading control using total protein staining. **(B)** microglial *Per-2* mRNA level in the late phase of the circadian cycle demonstrated as fold change vs. early phase in *Hmox1*^*fl/fl*^ and *LyzM-Cre-Hmox1*^*fl/fl*^ cells. Early and late phases in the circadian cycle were induced by different timing of the medium change. (*Hmox1*^*fl/fl*^ late vs. early phase *p* = 0.0003, *LyzM-Cre-Hmox1*^*fl/fl*^ late vs. early phase *p* = 0.0359, n = 6 per group). **(C)** microglial Cry-1 mRNA levels demonstrated as fold change vs. early phase in *Hmox1*^*fl/fl*^ and *LyzM-Cre-Hmox1*^*fl/fl*^ cells (*Hmox1*^*fl/fl*^ late vs. early phase *p* = 0.009, n = 5–6 per group). **(D)** The circadian difference in microglial *Per-2* mRNA expression demonstrated as fold change with carbon monoxide (CO) treatment (250ppm, 1 h) vs. control in *Hmox1*^*fl/fl*^ and *LyzM-Cre-Hmox1*^*fl/fl*^ cells (*Hmox1*^*fl/fl*^ late phase CO vs. control *p* = 0.0498, n = 6). **(E)** circadian difference in microglial Cry-1 mRNA expression demonstrated as fold change with CO treatment (250ppm, 1 h) vs. control in *Hmox1*^*fl/fl*^ and *LyzM-Cre-Hmox1*^*fl/fl*^ cells (*Hmox1*^*fl/fl*^ early phase CO vs. control *p* = 0.0188, n = 6) Used statistical analysis: one sample t-test; results presented as box blot with median, minimum and maximum. Statistically significant values were defined as *p* ≤ 0.05 (* *p* ≤ 0.05; ** *p* ≤ 0.01, *** *p* ≤ 0.001, **** *p* ≤ 0.0001)

To further explore the role of HO-1 in circadian regulation, we used primary microglial cells (PMG) derived from myeloid-specific HO-1-deficient mice (*LyzM-Cre-Hmox1*^*fl/fl*^) (Fig. 2A). Compared to *Hmox1*^*fl/fl*^ control PMG, HO-1-deficient microglia showed desynchronized *Cry-1* and *Per-2* expression (Fig. 2B *Hmox1*^*fl/fl*^*p* = 0.0003 and *LyzM-Cre-Hmox1*^*fl/fl*^*p* = 0.0359 & 2 C *Hmox1*^*fl/fl*^*p* = 0.009 and *LyzM-Cre-Hmox1*^*fl/fl*^*p* = 0.102). We furthermore wanted to explore the potential effect of CO exposure on circadian regulation to determine potential phases in the circadian cycle more susceptible to CO treatment. Therefore, PMGs in different circadian rhythms were exposed to 250ppm CO 1 h prior to harvest. We were able to demonstrate a CO-dependent lower *Per-2* expression in the late phase of the circadian cycle in *Hmox1*^*fl/fl*^ PMG compared to the air control group (Fig. 2D *Hmox1*^*fl/f*^ late phase *p* = 0.0498). Furthermore, CO exposure led to a lower *Cry-1* expression in the early phase, but not in the late phase compared to air exposure (Fig. 2E *Hmox1*^*fl/f*^ early phase *p* = 0.0188). In contrast, in HO-1 deficient microglia exogenous CO did not alter *Per-2* and *Cry-1* mRNA expression independent of the circadian timing (Fig. 2D & E).

### Mortality in HO-1 knockout after SAH is increased at ZT12 and can be rescued by CO but does not affect neurocognitive function

**Fig. 3:**
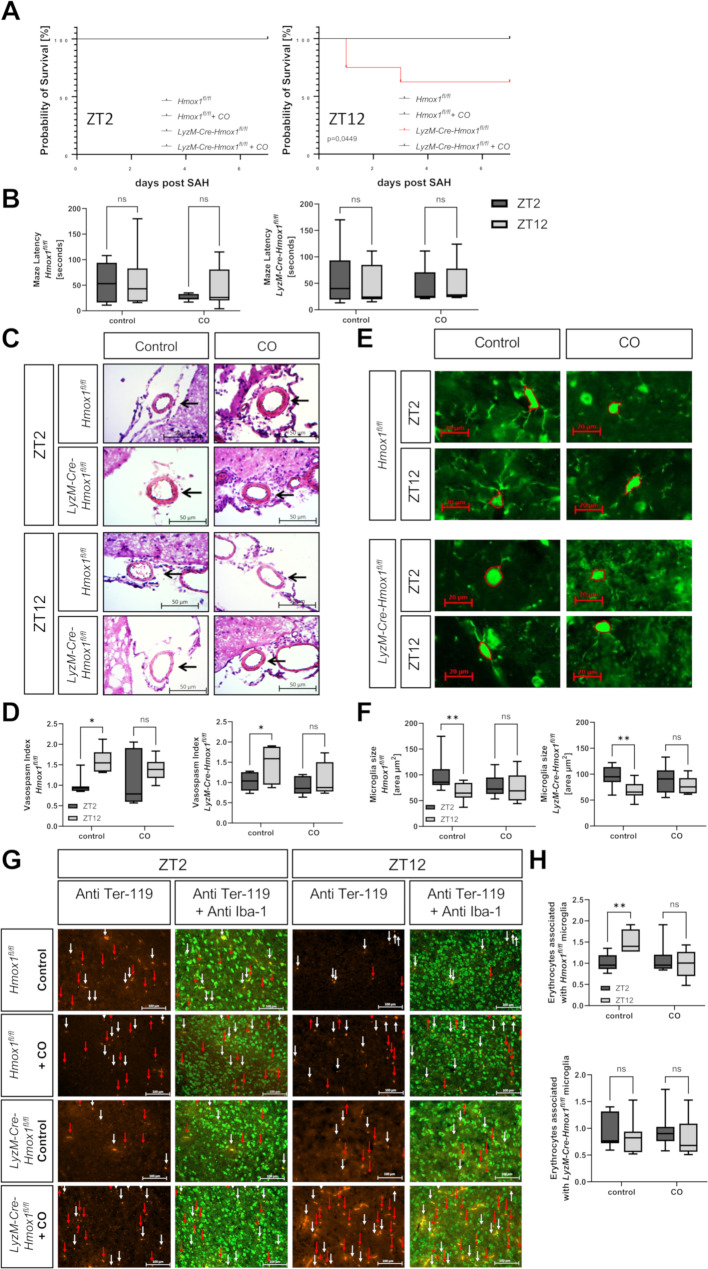
**(A-H):** Influence of circadian rhythm after SAH in vivo Analysis of *Hmox1*^*fl/fl*^ and HO-1 deficient *LyzM-Cre-Hmox1*^*fl/fl*^ mice. After blood injection, mice were air or carbon monoxide (CO) exposed daily (1 h); blood injection at ZT2 = 2nd hour and ZT12 = 12th hour of the 12-hour light cycle of the mice. **(A)** Survival 7 days post-SAH in *Hmox1*^*fl/fl*^ and *LyzM-Cre-Hmox1*^*fl/fl*^ air or CO exposed (ZT2 n = 6–7 and ZT12 n = 5–8, *p* = 0.0449). **(B)** Relative latency times ZT2 vs. ZT12 in the Barnes maze spatial memory paradigm of *Hmox1*^*fl/fl*^ and *LyzM-Cre-Hmox1*^*fl/fl*^ air or CO exposed on day 14 (7 days post-SAH) n = 5–7. **(C)** Hematoxylin/Eosin staining of the middle cerebral artery (MCA) 7 days post-SAH of *Hmox1*^*fl/fl*^ and *LyzM-Cre-Hmox1*^*fl/fl*^ controls and treated with CO at ZT2 and ZT12. **(D)** Vasospasm index (lumen radius [LR]/wall thickness [WT] normalized to *Hmox1*^*fl/fl*^ control ZT2, representative images in 4 C, n = 3–4 mice, 2–3 images per mouse, *Hmox1*^*fl/fl*^ control *p* = 0.035, *LyzM-Cre-Hmox1*^*fl/fl*^ control *p* = 0.038). **(E)** Representative microglia in the hippocampus; measurement of soma area; scale bars 20 μm, 7 days post-SAH. **(F)** Hippocampus (HC) microglia soma size (representative images in 4E, n = 3–4 mice; 2–4 images with microglia per mouse, *Hmox1*^*fl/fl*^ control *p* = 0.0046 and, *LyzM-Cre-Hmox1*^*fl/fl*^ control *p* = 0.0032). **(G)** For phagocytosis analysis, colocalization of immunofluorescent stained Ter-119 positive erythrocytes and Iba-1 stained microglia were analyzed; representative images of the analyzed brain region (base of the brain with proximity to the hypothalamic nuclei). White arrows show erythrocytes associated with microglia (colocalization of Ter-119 and Iba-1 signals) and red arrows show unassociated erythrocytes (Ter-119 signal alone). Scale bars 100 μm. (H) Erythrocytes associated with *Hmox1*^*fl/fl*^ or *LyzM-Cre-Hmox1*^*fl/fl*^ microglia normalized to *Hmox1*^*fl/fl*^ control ZT2. Analysis of phagocytosis (representative images in 4G; n = 3–4 mice with 2–4 images per mouse, *Hmox1*^*fl/fl*^ control *p* = 0.0062). Used statistical analysis: Log-rank (Mantel-Cox) test (Fig. 4A), two-way ANOVA with multiple comparison test; results presented as box blot with median, minimum and maximum. Statistically significant values were defined as *p* ≤ 0.05 (* *p* ≤ 0.05; ** *p* ≤ 0.01, *** *p* ≤ 0.001, **** *p* ≤ 0.0001)

SAH is a severe brain bleeding with following neurological sequelae and increased mortality [33], especially after high-grade SAH [34]. Observing the mortality rates after SAH with survival curves, no difference between *Hmox1*^*fl/fl*^ and *LyzM-Cre-Hmox1*^*fl/fl*^ mice with SAH induction at ZT2 could be noted (Fig. 3A). In contrast, *LyzM-Cre-Hmox1*^*fl/fl*^ mice with ZT12 blood induction exhibited increased mortality without CO treatment. *LyzM-Cre-Hmox1*^*fl/fl*^ mice showed a significantly lower survival rate at ZT12 (75% 1d post-SAH, 62.5% 3d after SAH; *p* = 0.0449), which could not be observed in the CO treated *LyzM-Cre-Hmox1*^*fl/fl*^ ZT12 mice, thus indicating a protective effect of exogenous CO.

To further explore the circadian dependency of the effect of CO after SAH, we analyzed the performance of mice after SAH on the Barnes Maze as an indicator of neurological and cognitive function. However, spatial memory function did not differ neither in *Hmox1*^*fl/fl*^ nor in *LyzM-Cre-Hmox1*^*fl/fl*^ mice regardless of the time point of SAH induction or the CO treatment (Fig. 3B, *Hmox1*^*fl/fl*^ control *p* = 0.9573 and CO *p* = 0.5835; *LyzM-Cre-Hmox1*^*fl/fl*^ control *p* = 0.8852and CO *p* = 0.9999).

### SAH at ZT2 increases vasospasm and inflammation following SAH

The lower survival rate of ZT12 *LyzM-Cre-Hmox1*^*fl/fl*^ mice seemed not to be connected to the cognitive brain function per se. Thus, we further investigated both the extent of vasospasm as well as inflammation post-SAH. The extent of vasospasm was significantly affected by the circadian phase (Fig. 3C and D). At ZT2, increased vasospasm for *Hmox1*^*fl/fl*^ (*p* = 0.035), as well as *LyzM-Cre-Hmox1*^*fl/fl*^ mice (*p* = 0.038), treated with air was detected compared to ZT12. CO treatment was able to reduce this circadian difference in *Hmox1*^*fl/fl*^mice (*p* = 0,4589) as well as *LyzM-Cre-Hmox1*^*fl/fl*^ mice (*p* = 0,6316).

The hippocampus is known for its high density of neurons and high susceptibility to injuries and inflammation [35–37]. Therefore, we analyzed microglia size at the hippocampus 7 days post-SAH showed the circadian dependency of their morphological features. Soma size was increased at ZT2 in *Hmox1*^*fl/fl*^ (*p* = 0.0046) and *LyzM-Cre-Hmox1*^*fl/fl*^ animals (*p* = 0,0032) (Fig. 3E and F). Treatment with exogenous CO decreased the difference between microglial soma size at ZT2 and ZT12 in *Hmox1*^*fl/fl*^ as well as in *LyzM-Cre-Hmox1*^*fl/fl*^leading to a similar microglia activation state (*Hmox1*^*fl/fl*^*p* = 0,9256; *LyzM-Cre-Hmox1*^*fl/fl*^*p* = 0,3548). Furthermore, *Hmox1*^*fl/fl*^ animals displayed significantly less association between Iba-1 and Ter119 positive signals at ZT2 compared to ZT12. Phagocytotic activity at ZT2 was therefore lower than at ZT12 (*p* = 0,0062) (Fig. 3G and H). This circadian-dependent difference diminished after CO treatment in *Hmox1*^*fl/fl*^ mice (*p* = 0,6228) and was also not detectable in HO-1 deficient *LyzM-Cre-Hmox1*^*fl/fl*^*mice* (control *p* = 0,7638; CO *p* = 0,6161).

In summary, induction of SAH at ZT2 led to more vasospasm, more neuroinflammation and less phagocytosis at ZT2 compared to ZT12 in *Hmox1*^*fl/fl*^. While *LyzM-Cre-Hmox1*^*fl/fl*^ mice displayed similar effects on vasospasm and neuroinflammation, these deficient mice did not show a difference in phagocytosis between ZT2 and ZT12, suggesting a role of HO-1 in this circadian dependency. Treatment with exogenous CO lead to an assimilation of the outcomes of SAH inducted at ZT2 and ZT12. These data suggest a crucial role for circadian timing in the development of neurological damage after SAH with a close relation to the HO-1-CO system.

### MCP-1 production is strongly induced in the CSF of SAH patients and correlates with Per-2 expression, SAH severity, hematoma size, the occurrence of delirium and worse outcome

**Fig. 4:**
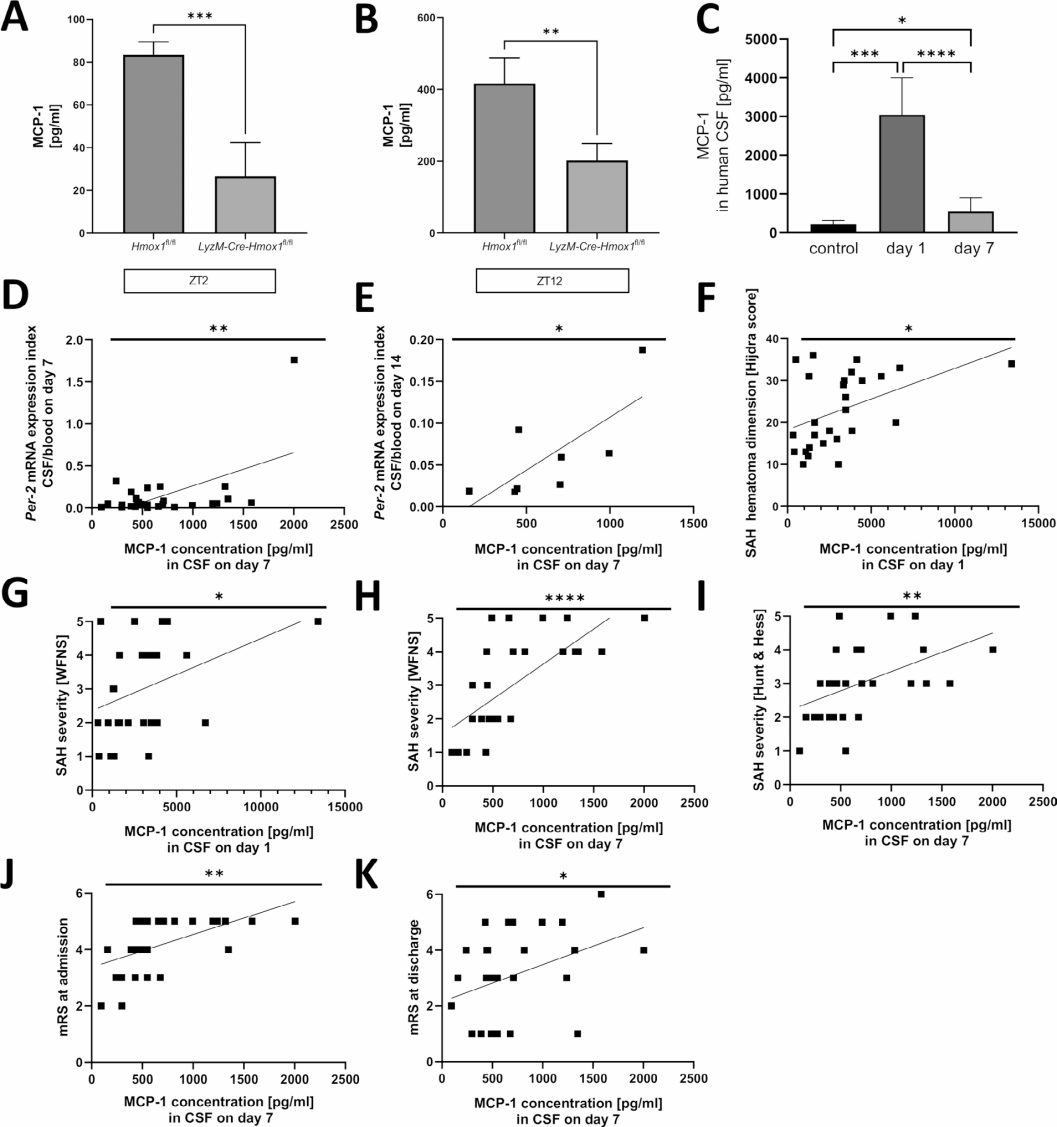
**(A-J)** Correlations between MCP-1, circadian patterns and neurological outcome humans **(A)** MCP-1 concentrations in culture medium at ZT2 (= 2 h after medium change) after 1 h treatment with 100 µl blood in PMG (*Hmox1*^*fl/fl*^ vs. *LyzMCre-Hmox1*^*fl/fl*^) measured with Cytometric Bead Array (CBA) analysis (n = 4, *p* = 0.0005). **(B)** MCP-1 concentrations in culture medium at ZT12 (= 12 h after medium change) after blood treatment in PMG (*Hmox1*^*fl/fl*^ vs. *LyzMCre-Hmox1*^*fl/fl*^) measured with Cytometric Bead Array (CBA) analysis (n = 4, *p* = 0.0024). **(C)** MCP-1 concentration in cerebrospinal fluid (CSF) in human controls, on day 1 and day 7 post-SAH (control vs. day 1 *p* = 0.0004 and day 7 *p* = 0.0214, day 1 vs. day 7 *p* < 0.0001). **(D)** MCP-1 concentration on day 7 in human CSF and correlation to Per-2 mRNA expression Index CSF/blood on day 7 (r^2^ = 0.3061, *p* = 0.0023). **(E)** MCP-1 concentration on day 7 in human CSF correlation to Per-2 mRNA expression Index CSF/blood on day 14 (r^2^ = 0.5445, *p* = 0.0366). **(F)** MCP-1 concentration on day 1 in human CSF correlation with SAH severity (Hijdra) (r^2^ = 0.1998, *p* = 0.0194). **(G)** MCP-1 concentration on day 1 in human CSF correlation with SAH severity (WFNS) (r^2^ = 0.1562, *p* = 0.0413). **(H)** MCP-1 concentration on day 7 in human CSF correlation with SAH severity (WFNS) (r^2^ = 0.4795, *p* = < 0.0001). **(I)** MCP-1 concentration on day 7 in human CSF correlation with SAH severity (Hunt & Hess) (r^2^ = 0.2346, *p* = 0.0078). **(J)** MCP-1 concentration on day 7 in human CSF correlation with mRS at admission (r^2^ = 0.2998, *p* = 0.0021). **(K)** MCP-1 concentration on day 7 in human CSF correlation with mRS at discharge (r^2^ = 0.1490, *p* = 0.0386). Statistical analysis by unpaired t-test **(A-B)**, Mann-Whitney test (C control vs. day 1 or day 7), Wilcoxon test (C day 1 vs. day 7) or simple linear regression and correlation **(D-K)**. Statistically significant values were defined as *p* ≤ 0.05 (* *p* ≤ 0.05; ** *p* ≤ 0.01, *** *p* ≤ 0.001, **** *p* ≤ 0.0001)

To investigate the translational significance of our preclinical experimental findings, we examined markers of neuroinflammation and circadian regulation in the CSF of SAH patients. Microglia play a crucial part in the neuroinflammatory response related to hemorrhage. Their phagocytic and inflammatory activity is determined by expressed cytokines like MCP-1. We aimed to explore changes in MCP-1 expression levels in the CSF of SAH patients as a marker for the extent of neuroinflammation. First, in order to explore how MCP-1 secretion by microglia is affected by HO-1 deficiency and the circadian rhythm after blood exposure in vitro, we analyzed MCP-1 concentrations two hours after medium change (ZT2) and 12 h after medium change (ZT12) in control microglia and HO-1 deficient microglia supernatant after blood exposure. Interestingly, in the ZT2 as well as the ZT12 group, the levels of secreted MCP-1 were significantly lower in HO-1 deficient microglia compared to control microglia, indicating an influence of HO-1 on MCP-1 production (Fig. 4A and B; ZT2 *Hmox1*^*fl/fl*^ vs. *LyzM-Cre-Hmox1*^*fl/fl*^*p* = 0.0005, ZT12 *Hmox1*^*fl/fl*^ vs. *LyzM-Cre-Hmox1*^*fl/fl*^*p* = 0.0024).

To further examine the role of MCP-1 in human SAH, we measured MCP-1 concentration in the CSF on day 1 and day 7 after SAH compared to non-hemorrhage CSF controls. We found a distinct time kinetic of MCP-1 concentration in the CSF with the highest concentration on day 1 with a significant decrease on day 7 (*p* < 0.0001). (Fig. 4C). MCP-1 concentration in control CSF differed significantly from MCP-1 concentration on day 1 (*p* = 0.0004) or on day 7 (*p* = 0.0214) after SAH.

To analyze a potential link between MCP-1 secretion and circadian regulation, we measured *Per-2* expression levels after SAH and correlated these findings with MCP-1 concentrations. Per-2 expression levels 7 days (Fig. 4D, r^2^ = 0.3061, *p* = 0.0023) and 14 days (Fig. 4E, r^2^ = 0.5445, *p* = 0.0366) after SAH were positively correlated with MCP-1 concentration on day 7. We next aimed to explore whether MCP-1 might serve as an outcome predictor in the SAH patient collective. High MCP-1 concentration in the CSF was positively correlated with hematoma dimension quantified by the Hijdra sum score classification (Fig. 4F, MCP day 1, r^2^ = 0.1998, *p* = 0.0194), by the WFNS score (Fig. 4G, MCP-1 day 1, r^2^ = 0.1562, *p* = 0.0413; Fig. 4H, MCP-1 day 7, r^2^ = 0.4795, *p* = < 0.0001) and by the Hunt & Hess grade (Fig. 4I, MCP-1 day 7, r^2^ = 0.2346, *p* = 0.0078). Regarding functional neurological prospection and outcome, MCP-1 concentration on day 7 correlated significantly with unfavorable mRS at admission (Fig. 4J, r^2^ = 0.2998, *p* = 0.0021) and discharge (Fig. 4K, r^2^ = 0.1490, *p* = 0.0386). Additional File 2 shows non-significant correlations between MCP-1 on day 1 with *Per-2* on day 1, 7 and 14 and MCP-1 on day 7 with *Per-2* on day 1and hematoma size, respectively.

In summary, MCP-1 concentration showed a distinctive time pattern in both in vivo and in *vitro* studies, indicating a circadian regulation with HO-1 as a regulatory factor. Furthermore, we were able to identify MCP-1 as an outcome predictor with a high expression level correlating with increased SAH severity and worse neurological outcome.

## Discussion

In this combined in vitro and in vivo study, we were able to detect a circadian regulation of HO-1 protein expression in microglia with higher HO-1 protein expression in the late phase of the circadian cycle. This circadian regulation of HO-1 protein expression was impaired after exposure to blood.

HO-1 plays an important role in the degradation of heme by converting heme into iron, biliverdin and carbon monoxide. As the inducible isoform, HO-1 is upregulated after exposure to blood and it plays an essential role in the decrease of neuronal cell death, clearance of cerebral blood, prevention of vasospasm and attenuation of impaired cognitive function [16, 38–40]. In SAH, heme-induced neuroinflammation as well as toxicity of blood-degradation products leads to neuronal injury [41].

In our previous study, we were able to show that the presence of peripheral HO-1 competent myeloid cells did not change the pathological phenotype attributable to HO-1 deficient microglia of the moue brain [16]. Therefore, by using HO-1 deficient microglia and mice for loss of function studies, we were directly able to show the relevance of this enzyme and its link to the cell’s circadian state in the microglial and SAH context. However, we did not differentiate between central resident myeloid cells (microglia) and potentially invading peripheral myeloid cells regarding their distinct pathophysiological role in our in vivo model, as the tissue-specific *HO-1* knockout applied to all myeloid cells and the amount of peripheral cell invasion after SAH was not quantified in the current project. Even though a link between CO as the end product of HO-1-mediated heme metabolism and the molecular regulation of circadian rhythm has been demonstrated [25, 26], our data provides evidence that the maintenance of circadian rhythmicity in microglia depends on the presence of HO-1. HO-1 deficiency led to a disturbed circadian rhythm and an impaired CO-induced down-regulation of *Per-2* in the late phase of the circadian cycle, thus indicating a potential HO-1-dependency of the CO-mediated effect on circadian rhythmicity. The fact that exogenous CO did not alter *Per-2* expression in HO-1 deficient microglia confirms the established concept that depending on the model and extent of injury, exogenous CO cannot compensate endogenous HO-1 depletion. Additionally, HO-1 deficiency led to altered Per-2 expression in the late phase but altered Cry-1 expression in the early phase. This indicates that HO-1 plays a crucial role on multiple levels in the complex circadian timing. The precise mechanism behind these alterations remains to be investigated with additional research.

While we found signs for increased vasospasm and enhanced inflammation at ZT2, acute mortality was significantly higher in mice with injury at ZT12 in mice with microglial HO-1 deficiency. This alleged inconsistency in circadian dependency of neuronal injury could possibly be explained by the temporal pathophysiology of SAH, although without further mechanistic studies this explanation remains speculative: During the acute phase within the first 24 h post hemorrhage, neuronal damage depends on the extent of intracranial pressure elevation, cerebral circulatory disturbance, cortical depression and brain edema. Decreased microglial reactivity at ZT12, as observed in our model, might hinder the body’s acute response to SAH-induced injury, leading to fatal neuronal damage.

In contrast, the pathophysiological features during the subacute phase of SAH-induced injury up to three days after hemorrhage are mainly related to hematoma removal, heme metabolism and blood component cytotoxicity. Here, overshooting inflammation related to increased microglial reactivity at ZT2 might contribute to increased neuronal damage at this time point. Therefore, one can speculate that the initial microglia activation is the crucial part for survival post SAH. Nevertheless, while interpreting these data it should be taken into account that the ZT12 data of HO-1 deficient mice only represent the healthier, surviving specimen. Naturally, maze latency or CO treatment effect data could not be obtained from animals that have died early in the course of the experiment.

Interestingly, HO-1 deficient mice had a higher survival rate with CO treatment then without after SAH induction at ZT12. These results are in line with our findings featuring high expression levels of HO-1 in vitro in the late circadian phase and with previous results on *HO-1* expression during the circadian cycle [18, 42]. A lack of HO-1 at times of normally high expression increases acute neuronal damage.

In contrast to the higher survival rates of HO-1-deficient mice at ZT2, control mice after SAH at ZT2 displayed increased vasospasm, more activated microglia at the hippocampus and less phagocytic activity compared to ZT12. Animals treated with CO did not show this difference between ZT2 and ZT12, suggesting that CO exerts protective effects on long-term cerebral damage after SAH at the early circadian phase only. These findings match the results of previous studies that demonstrated worse neurological outcomes at ZT2, the beneficial effects of CO on circadian rhythm [18] and the potential of CO to restore circadian rhythm [43].

Prior research was able to define Per-2 as a diagnostic marker for prognostication of delirium and functional outcome after SAH [28] and after head trauma [44]. Our in vitro and correlative patient data suggest that both circadian regulatory proteins and inflammatory markers such as MCP-1 display concordant changes in expression levels, indicating circadian dependency of the inflammatory response, which in turn is regulated by HO-1 during the complex interaction between neuronal cells and microglia. This is well in line with in vivo data for other organs, demonstrating this dependency in murine liver tissue [43]. As a result, endogenous HO-1 deficiency would present a disadvantage due to desynchronized *Per-2* expression and altered inflammatory response after hemorrhage. Furthermore, exogenous CO application as a potential therapeutic approach seems to be dependent on the timing of application leading to differences in the inducibility of HO-1, *Per-2* expression and the inflammatory response.

To provide a link between the in vitro and human data, we analyzed MCP-1 production of microglia at ZT2 and ZT12 and MCP-1 concentrations in human CSF post-SAH. As MCP-1 was measured in the culture medium, we were not able to show a direct circadian regulation of the MCP-1 level, as they would naturally accumulate in the medium over time. However, we were able to show that the MCP-1 level after blood exposure was lower in HO-1-deficient cells. In speculation about the relevance of these findings, we propose that MCP-1 as one of the main cytokines responsible for the migration and infiltration of monocytes and macrophages would be beneficial in the first hours after SAH. In line with this, Prolo et al. demonstrated a migration-stimulating effect of MCP-1 in BV-2 cells [45]. In vivo and human data suggest a specific time course of cerebral MCP-1 expression [46, 47]. However, the outcome-specific relevance of the quantity of MCP-1 remains unclear. Our clinical data demonstrates that MCP-1 production was physiologically increased and strongly induced after SAH with a peak on day 1 post-SAH and is therefore in accordance with previous works showing increased MCP-1 after SAH [47]. We found a correlation between sustained MCP-1 elevation on day 7 after hemorrhage and unfavorable clinical outcomes. Elevated MCP-1 levels have been described as a predictive marker after SAH [48]. Referring back to the in vitro findings, HO-1 deficient microglia showed decreased MCP-1 production. Future research must establish a mechanistic link of these findings to the pathological phenotype observed in the murine SAH model with regard to HO-1 deficiency, CO exposure and circadian dependency of outcome. Our data confirm a possible dual function of MCP-1 and suggest that sustained MCP-1 could act as a surrogate for excess neuroinflammation.

## Conclusion

Taken together, we were able to demonstrate a novel role for HO-1 in that it critically determines circadian regulation in microglia with a concomitant change in their ability to respond to an inflammatory stimulus such as hemorrhage. Using HO-1 deficient primary cells, we were furthermore able to demonstrate the importance of HO-1 for circadian synchronization and inflammatory cytokine production such as MCP-1. The functional in vivo relevance of these findings was demonstrated in a corresponding murine SAH model and correlative patient data. In the future, the circadian dependency of these effects might be an important consideration in the planning of studies exploring the therapeutic potential of interventions targeting the HO-1/CO system and the neuroinflammatory response following SAH. Furthermore, due to the vital role of HO-1 in the reaction to SAH and its circadian dependency, our findings could be important in future research regarding clinical outcome and timing of treatment.

### Electronic supplementary material

Below is the link to the electronic supplementary material.

Supplementary Material 1: Raw Image Files to Figure 1B and Figure 2A.

Supplementary Material 2: Supplemental Figure 1: Correlations between MCP-1, Per-2 expression and hematoma size post SAH

## Data Availability

The datasets used and/or analyzed during the current study are available from the corresponding author upon reasonable request.

## Abbreviations

°C: Celsius
µg: µgram
µl: µliter
µm: µmeter
BMAL-1: Basic Helix-Loop-Helix ARNT Like 1
BSA: Bovine Serum Albumin
CD11b: Cluster of Differentiation 11b
cDNA: complementary Deoxyribonucleic Acid
CLOCK: Circadian Locomotor Output Cycles Kaput
Cm: centimeter
CNS: Central Nervous System
CO: Carbon monoxide
CO_2_: Carbon dioxide
Cry: Cryptochrome
CSF: Cerebrospinal Fluid
CT: computer tomography
DAPI: 4′,6-diamidino-2-phenylindole
ECL: Enhanced Chemiluminescence
EDTA: etheylenediaminetetraacetic acid
EVD: external ventricular drain
FACS: fluorescence-activated cell sorting
FBS: Fetal Bovine Serum
Fl: flox
H: hour(s)
HC: Hippocampus
Hmox1: Heme oxygenase-1 (mouse strain genetic information)
HO-1: Heme oxygenase-1 (gene / protein)
Iba-1: Ionized calcium-binding adapter molecule 1
ICU: intensive care unit
IL: interleukin
IP-10: interferon-gamma induced protein 10 kD
Kg: kilogram
LR: lumen radius
Lyz: Lysozyme
MCA: Middle Cerebral Artery
MCP-1: Monocyte Chemoattractant Protein-1
M-CSF: Macrophage Colony-Stimulating Factor
Mg: milligram
MIG: Monokine induced by Interferon Gamma
Min: minute
Mm: millimeter
mRS: modified Rankin Scale
Na-Azide: Sodium-Azide
nM: nanomolar
NPAS-2: Neuronal PAS domain-containing protein 2
Ns: non-significant
NuDESC: Nursing Delirium Screening Scale
O_2_: Oxygen
PBS: Phosphate buffered saline
PCR: Polymerase chain reaction
PE-Cy7: phycoerythrin-cyanin7
Per: Period
PFA: Paraformaldehyde
PMG: Primary Microglia
Ppm: parts per million
PVDF: polyvinylidene difluoride
RANTES: Regulated upon Activation, Normal T Cell Expressed and Secreted
RIPA: radioimmunoprecipitation assay
RNA: Ribonucleic acid
Rpl13a: Ribosomal protein L13a
Rplp0: ribosomal protein, large, P0
Rpm: rotations per minute
SAH: Subarachnoidal Hemorrhage
SCN: Suprachiasmatic Nucleus
SD: Standard deviation
SDS: Sodium dodecyl sulfate
TBS: Tris-Buffered Saline
UV: Ultra Violet
vs.: versus
W: watt
WFNS: World Federation of Neurosurgical Societies
WT: wall thickness
ZT: Zeitgeber time
ΔCT: Cycle Threshold
