# Supplementary figures and images for: Circadian dependency of microglial heme oxygenase-1 expression and inflammation determine neuronal injury in hemorrhagic stroke
Source: J Inflamm (Lond). 2023 Dec 16;20:43. doi: 10.1186/s12950-023-00371-w (PMC10725034; doi:10.1186/s12950-023-00371-w)

Figure 1B

anti-HO-1

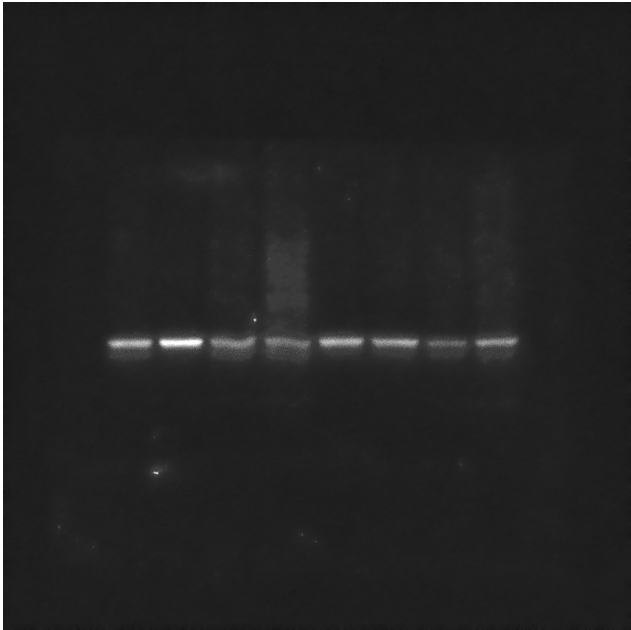

total protein

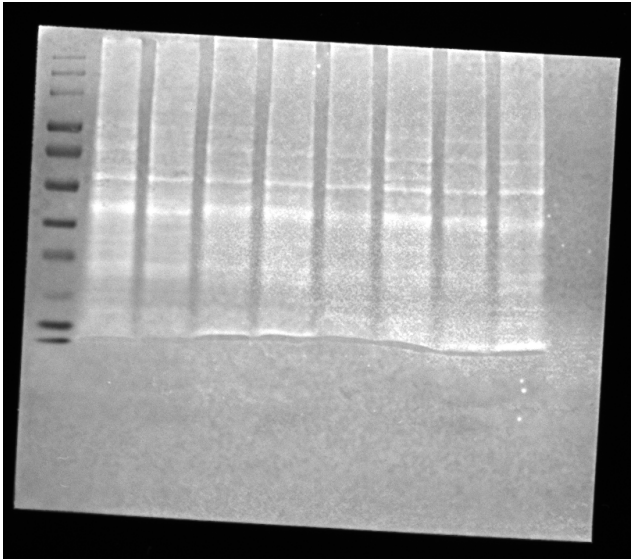

Figure 2A

anti-HO-1

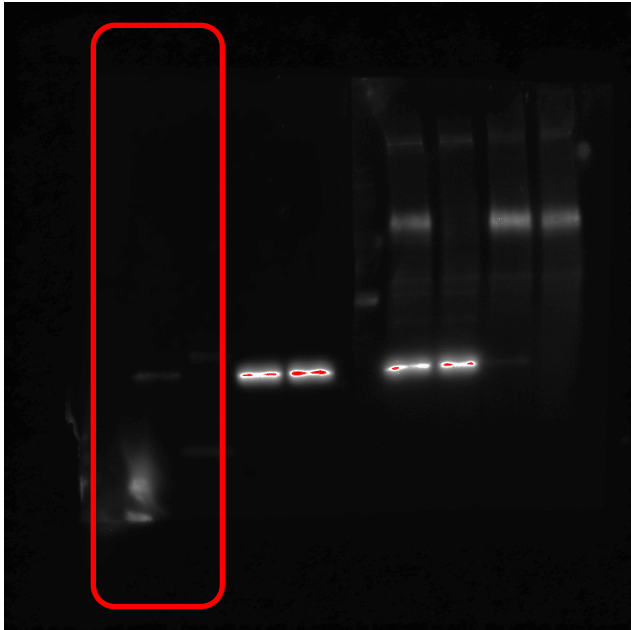

(anti-HO-1 invert)

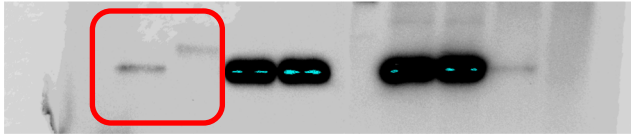

total protein

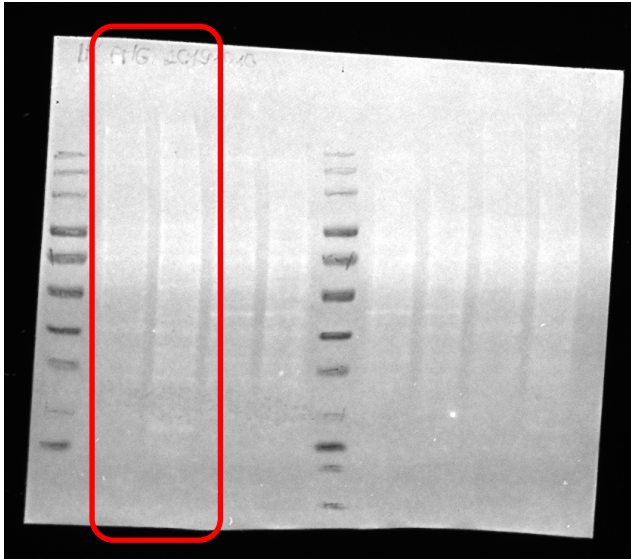

Supplement: Supplementary file 1 — Supplementary Material 1: Raw Image Files to Figure 1B and Figure 2A. [file 12950_2023_371_MOESM1_ESM.pdf]
