# Supplementary material for: Circadian dependency of microglial heme oxygenase-1 expression and inflammation determine neuronal injury in hemorrhagic stroke
Source: J Inflamm (Lond). 2023 Dec 16;20:43. doi: 10.1186/s12950-023-00371-w (PMC10725034; doi:10.1186/s12950-023-00371-w)
Supplement: Supplementary file 2 — Supplementary Material 2: Supplemental Figure 1: Correlations between MCP-1, Per-2 expression and hematoma size post SAH [file 12950_2023_371_MOESM2_ESM.pdf]

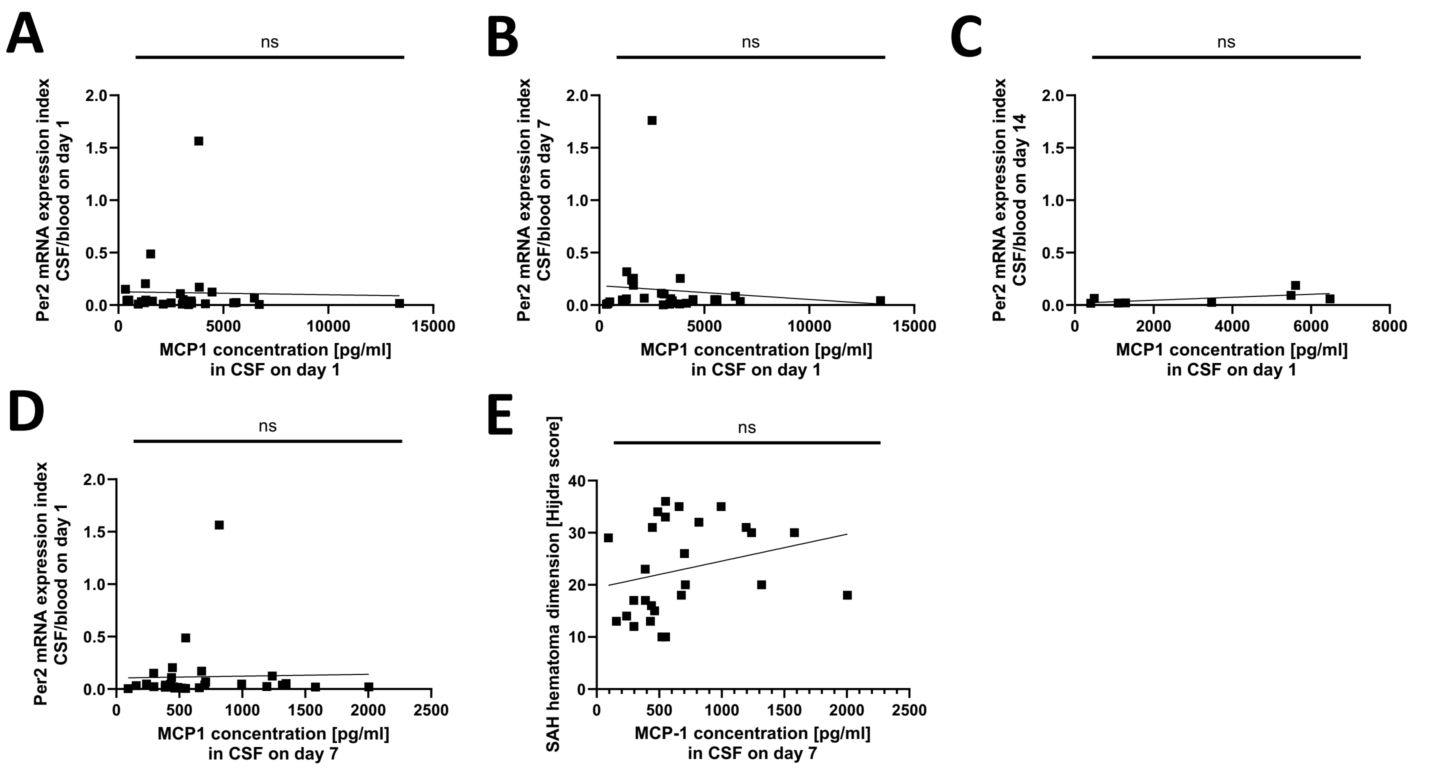

Supplemental Figure 1: Correlations between MCP-1, *Per-2* expression and hematoma size post SAH

(A) MCP-1 concentration on day 1 in human CSF and correlation to Per-2 mRNA expression Index CSF/blood on day 1 (r^2=0,0006407, p=0,8963). (B) MCP-1 concentration on day 1 in human CSF correlation to Per-2 mRNA expression Index CSF/blood on day 7 (r^2=0,01118, p=0,5923). (C) MCP-1 concentration on day 1 in human CSF correlation to Per-2 mRNA expression Index CSF/blood on day 14 (r^2=0,3857, p=0,1003). (D) MCP-1 concentration on day 7 in human CSF correlation to Per-2 mRNA expression Index CSF/blood on day 1 (r^2=0,0007193, p=0,8902). (E) MCP-1 concentration on day 7 in human CSF correlation with SAH severity (Hijdra) (r^2=0,06901, p=0,1856).
